# Knowledge translation strategies to improve the use of evidence in public health decision making in local government: intervention design and implementation plan

**DOI:** 10.1186/1748-5908-8-121

**Published:** 2013-10-09

**Authors:** Rebecca Armstrong, Elizabeth Waters, Maureen Dobbins, Laurie Anderson, Laurence Moore, Mark Petticrew, Rachel Clark, Tahna L Pettman, Catherine Burns, Marjorie Moodie, Rebecca Conning, Boyd Swinburn

**Affiliations:** 1 Jack Brockhoff Child Health and Wellbeing Program, McCaughey VicHealth CentreMelbourne School of Population and Global Health, University of Melbourne, Level 5/207 Bouverie Street, Carlton, Victoria, Australia; 2 School of Nursing, McMaster University, Main Street West, Hamilton, Ontario, Canada; 3 Department of Epidemiology, School of Public Health, University of Washington, Seattle, USA; 4 Cardiff Institute of Society and Health, School of Social Sciences, Cardiff University, 1-3 Museum Place, Cardiff, Wales, UK; 5 London School of Hygiene and Tropical Medicine, University of London, Keppel St, London, UK; 6 The Center of Excellence in Intervention and Prevention Science, Melbourne, Australia; 7 Population Health Strategic Research, Deakin University, 221 Burwood Highway, Burwood, Victoria, Australia; 8 Deakin Health Economics, School of Health and Social Development, Deakin University, Burwood Highway, Burwood, Victoria, Australia

**Keywords:** Knowledge translation, Evidence, Public health, Decision-making

## Abstract

**Background:**

Knowledge translation strategies are an approach to increase the use of evidence within policy and practice decision-making contexts. In clinical and health service contexts, knowledge translation strategies have focused on individual behavior change, however the multi-system context of public health requires a multi-level, multi-strategy approach. This paper describes the design of and implementation plan for a knowledge translation intervention for public health decision making in local government.

**Methods:**

Four preliminary research studies contributed findings to the design of the intervention: a systematic review of knowledge translation intervention effectiveness research, a scoping study of knowledge translation perspectives and relevant theory literature, a survey of the local government public health workforce, and a study of the use of evidence-informed decision-making for public health in local government. A logic model was then developed to represent the putative pathways between intervention inputs, processes, and outcomes operating between individual-, organizational-, and system-level strategies. This formed the basis of the intervention plan.

**Results:**

The systematic and scoping reviews identified that effective and promising strategies to increase access to research evidence require an integrated intervention of skill development, access to a knowledge broker, resources and tools for evidence-informed decision making, and networking for information sharing. Interviews and survey analysis suggested that the intervention needs to operate at individual and organizational levels, comprising workforce development, access to evidence, and regular contact with a knowledge broker to increase access to intervention evidence; develop skills in appraisal and integration of evidence; strengthen networks; and explore organizational factors to build organizational cultures receptive to embedding evidence in practice. The logic model incorporated these inputs and strategies with a set of outcomes to measure the intervention’s effectiveness based on the theoretical frameworks, evaluation studies, and decision-maker experiences.

**Conclusion:**

Documenting the design of and implementation plan for this knowledge translation intervention provides a transparent, theoretical, and practical approach to a complex intervention. It provides significant insights into how practitioners might engage with evidence in public health decision making. While this intervention model was designed for the local government context, it is likely to be applicable and generalizable across sectors and settings.

**Trial registration:**

Australia New Zealand Clinical Trials Register ACTRN12609000953235.

## Background

Evidence-informed decision-making (EIDM) involves integrating the best available research evidence with contextual factors including community preferences, local issues (*e.g.*, health, social), political preferences, and public health resources [1-3]. EIDM therefore considers research evidence as one form of a range of sources of evidence that are used to inform policy and practice [2,4]. EIDM can be applied in a range of decision-making contexts (including policy development, implementation, and evaluation [5]). The benefits of EIDM include adoption of the most effective and cost-efficient interventions [6], minimized harm to people and communities [7-9], and better health outcomes for individuals and communities [10-12].

However, in order for EIDM to operate efficiently and effectively, a series of mechanisms are required: research evidence needs to be conceptualized, conducted, and communicated in a way that is meaningful to decision makers [13,14]; research evidence needs to be accessed, assessed, and appropriately applied [15] by decision makers within a complex political system; and researchers and decision makers need to work in partnership to fund and conduct research that addresses key policy questions. It is clear that decision makers are under increasing pressure to ensure their decisions are ‘evidence-based’ [16,17] but significant barriers have been identified. These include absence of personal contact between researchers and policy makers and practitioners, lack of time and resources, organizational structures, and decision-making processes, timeliness of research, poor quality or limited availability of research, poor reporting of research, and political influence [13,15,18-20].

To address these barriers, a range of strategies, often conceptualized as knowledge translation (KT), have been developed, described, and in some instances, implemented, particularly in clinical and health services contexts. KT is informed by and builds upon conceptual understandings of the translation of research into practice, for which key theories include diffusion, dissemination, and implementation. Diffusion efforts are generally passive while dissemination is a more active strategy to promote the spread of particular ideas [21]. Implementation refers to systematic efforts to encourage adoption of evidence and knowledge by overcoming barriers [22]. This article is concerned with KT strategies that seek to support implementation and describes an approach to the implementation of KT strategies within local governments (LGs). KT strategies range between researcher-focused interventions (to support the dissemination of research findings), decision maker-focused interventions (to change practices and behaviors related to the integration of research evidence into decision-making processes), and interventions designed to create partnerships between researchers and decision makers (where questions of mutual interest are identified, research is conducted in partnership, and the research is used to inform policy-level decisions) [23].

Despite the interest and advocacy for KT activities, there has been limited evaluation of their impact on research use in practice and policy and ultimately, EIDM in public health. The implementation of KT strategies to date has tended to focus on individual behavior change of decision makers (*e.g.*, improving access to research to ensure decision makers know where and how to access it) [24]. However, it is increasingly acknowledged that strategies to boost user capacity to access and use research will have limited effect unless organizational and system-wide barriers are addressed [15], where the reach goes beyond individuals. This particularly likely to be the case in public health where support systems are often not available [25], for example EIDM processes are generally not institutionalized within organizational processes. There are also likely to be staff from diverse professional backgrounds and as a result varying levels of skill and confidence in EIDM.

Given the distinct need for further intervention research examining the effectiveness of KT strategies for public health decision-making, we explored the interest of the LG sector. There was strong support for participating in an intervention to explore the effectiveness of KT strategies. The KT intervention, named Knowledge Translation for Local Government or ‘KT4LG,’ was developed with the 79 LG locations and implemented within the research context of an exploratory cluster randomized controlled trial. The evaluation of this intervention represents a KT implementation study and was registered with the international clinical trials portal (Australian New Zealand Clinical Trials Register ACTRN12609000953235). The study protocol for the design and methods for the mixed method evaluation was published prior to commencement [26].

The KT4LG intervention aimed to increase the use of research evidence to support EIDM for public health in LGs. The purpose of this paper is to describe the design of, and implementation plan for, the intervention. We conducted four preliminary studies to produce findings to contribute into the development of the logic model. The studies aimed to explore:

1. What KT strategies are described in the literature in public health and more broadly and what do we know about their effectiveness?

2. What types of evidence are used by Victorian LGs, and how is it used within the context of decision making?

3. What KT strategies are most applicable to Victorian LGs to support EIDM?

A program logic was developed from findings of these studies to articulate and describe the intervention components, intended outcomes of the intervention, and possible evolutions at organizational- and system-levels [27].

### Context

In Australia, LGs represent the third tier of government and are responsible for community-level issues, controlled by Acts of state or territory parliaments. Broadly, their responsibilities include: planning and building; roads and parking; health services (for example, food safety, prevention of spread of infectious disease); people services (for example, maternal and child health); waste management; animal management; recreation and culture; and local laws [28]. The political and geographical context for this study is Victoria Australia, where state-level legislation requires LGs (councils) to have an evidence-based municipal public health plan [29]. Section five of the Act states that decisions should be based on the best available evidence to ensure effective and efficient use of resources. Obesity prevention was selected as the public health topic for the study, harnessing the capacity of LGs to intervene by affecting policy and regulatory change in food and physical activity environments [30,31] and enabling illustration of the KT program components in the context of a national health priority area. Through broader socio-environmental determinants, obesity prevention is an indirect priority area within LGs, with many councils working on food security, open space for physical activity, and public transport connections (*e.g.*, [32,33]) More recently, LGs in Australia have assumed key responsibilities for healthy eating and physical activity implementation through nationally-funded grants (http://www.healthyactive.gov.au/internet/healthyactive/publishing.nsf/Content/healthy-communities). In this context, the KT intervention was designed to address known barriers to research evidence uptake and to identify strategies that are effective in improving the degree to which research evidence is accessed, assessed, adapted and applied by decision makers.

The purpose of this paper is to describe the design of and implementation plan process for a KT intervention for public health decision making in LGs (the KT4LG study). Documenting the design and implementation of KT interventions is particularly important given that there is limited research but increasing interest in the application of KT to public health.

## Methods

This section describes the methods and provides a broad overview of results of the studies that informed intervention design and the implementation plan. This differs from a traditional methods section, but we feel it is important to document these studies to demonstrate their impact on intervention design and implementation. These preliminary studies included a review of theoretical and narrative literature, a systematic review of the effects of KT strategies [23]; a survey of LGs in Victoria, and a qualitative study exploring decision-making processes in Victorian LGs [5]. A concurrent triangulated design that treated each component (or study) relatively equally was applied. Data from each study were therefore collected concurrently, analyzed separately, then findings compared and synthesized for interpretation [34]. This enabled inferences to be made about the overall potential for conceptualization, and application, of KT in public health decision making in LGs.

### Study 1a: literature review

To understand potential barriers and facilitators, types of KT strategies and theoretical perspectives described and applied in contexts relevant to public health decision-making, we conducted a scoping review of the literature, including peer reviewed and grey literature. The search comprised a range of electronic databases and online sources (Medline, CINAHL, APAIS, PsychInfo, Web of Science, Google, and Google Scholar). Search terms included derivatives of translation, exchange, transfer, utilization, mobilization, evidence-based, evidence informed, public health, diffusion of innovations, knowledge management, decision making, policy. This provided insight into how EIDM might occur in public health decision-making contexts, and revealed gaps in the literature particularly in relation to theoretical models to guide KT intervention design.

Barriers to EIDM have been well documented, however the literature is primarily relevant to clinical and health service decision-makers. Of relevance to public health, the available literature suggests that issues include an unsupportive culture, lack of experience in assessing evidence, staff turnover, information overload, and lack of actionable messages [24]. Facilitators and influences have also been identified in the literature and include discussion on the influences of policy and context and decision-making factors on the evidence-informed process [4]. Understanding the barriers and the internal and external decision-making contexts [35] was crucial in mapping the varying levels that KT interventions could operate. Research utilization theory describes types of evidence use: instrumental, conceptual, and symbolic [36-38]. The extent to which instrumental use (the direct application of research) occurs or can be measured in the policy context has been challenged [39].

The literature identified the broad range of interventions characterized as supporting EIDM, albeit largely applied in clinical and health service settings, including strategies that aim to push evidence towards decision makers (*e.g.*, systematic reviews, clearinghouses), strategies that aim to encourage decision makers to seek out evidence (*e.g.*, incentives and rewards, facilitation) and strategies that aim to encourage the interaction between researchers and decision makers (*e.g.*, communities of practice, research partnerships). Studies with a focus on KT conducted in clinical medicine and allied health have traditionally focused on behavior change outcomes [40,41]. These are appropriate measures in these contexts where professional practice is overseen by clinical guidelines and where action (for example, prescribing or treatment choice) is more easily definable and measurable. However, public health decision-making is more complex [42]. While evidence may be considered within the decision-making process, but it may be difficult to identify its relative contribution to the final decision [39].

In order to determine an appropriate theoretical framework for our intervention, it was necessary to seek examples from the literature. Diffusion of innovations theory has been applied to other KT research studies to help explore how ‘innovations’ or policy ideas spread among individuals and organizations. Diffusion theory is useful in helping to identify how innovations spread within organizations, and in doing so is important in identifying points at which strategies/interventions to increase research use could be introduced. We also considered the context and system within which LGs operate, and the decision-making influences that exist within these structures. Discussions within the literature have also recently highlighted the context for KT recognising that diffusion and dissemination processes and relationships are shaped and embedded through structures [43-45].

### Study 1b: systematic review

Systematic reviews are important syntheses of the current state of best available evidence. They are also valuable in terms of identifying research gaps [46]. A systematic review of the effectiveness of KT interventions for public health decision makers and managers was conducted between 2009 and 2010. It is not possible to report here the extensive methodology, however further information has been reported elsewhere [23,44] and is available from the authors on request. The results of the review confirmed our expectations that there is limited evidence of the effectiveness of KT strategies in public health settings [5,26]. At the time of the review, only one controlled study had been reported globally [47], which found that targeted messages show potential for increasing research utilization. The study explored the role and effectiveness of a knowledge-translation intervention in 108 public health settings. The intervention that was evaluated included three components (each a separate study arm): access to an online registry of systematic reviews and associated summaries (‘website’); website plus tailored messages; and website plus tailored messages plus interaction with a knowledge broker (KB). The intervention duration was one year, and the hypotheses were that public health departments exposed to tailored messages and KB would report greater EIDM than those exposed to the website only. It was found that tailored messages ‘pushed’ to the right individuals (*e.g.*, decision makers), in an organization that was supportive of evidence use, led to positive outcomes. The KB did not appear to be effective, but it was noted to be potentially useful where the culture for evidence use was low. This may suggest a need for externally-driven capacity building for individuals (*e.g.*, skills, confidence), where the organizational culture is not supportive of evidence use. In sum, the systematic review of international published and unpublished literature found only one study, and thus limited evidence to explain what interventions facilitate evidence use in decision-making processes; no published information from a LG context; and no literature from the Australian context.

### Study 2: EviDenT: a statewide survey

Given the lack of literature describing public health engagement with evidence and a lack of intervention design to support EIDM within this context, the EviDenT (Evidence-informed Decision-making Tool) was developed to survey LGs across the state of Victoria. Informed by previous tools [48,49], the EviDenT incorporated three core domains based on the findings from study one; access to evidence, confidence in using evidence, and organizational culture for EIDM. Other questions focused on EIDM skills, the use and influence of a range of sources of evidence, and barriers and facilitators to EIDM. As will be reported elsewhere, all LGs were invited to participate in the survey and were asked to nominate up to four staff members who worked in areas relevant to public health. Analyses of the survey data (n = 135; response rate 75%) revealed emerging factors and strategies related to how decisions were being made within LGs, and how influential evidence could be. Importantly, the survey also identified potential KT strategies suggested by LG respondents that may be useful in the LG context, including: strategies to facilitate organizational change; improvements in access to evidence; and opportunities to participate in workforce development to build skills in EIDM. These suggestions would help to design the intervention. Improvements to time and resources available to practice EIDM were highlighted, and training was frequently reported as necessary to support EIDM, for example to develop skills in searching for, appraising trustworthiness of, and applying research evidence. Access to current research was identified, including access to research databases (which most LGs did not subscribe to). Improvement of organizational culture emerged, as evidence searching or review was not considered to be a necessary function. Time and additional resources were necessary to support EIDM in terms of accessing and reading research and/or human resources to perform this function. Respondents mentioned the need for more accessible information (research evidence summaries, regular bulletins, and cross-agency initiatives). A thorough report of the results of the EviDenT survey will be published elsewhere. A copy of the survey is available on request.

### Study 3: qualitative study: mechanisms to support EIDM

Although EviDenT provided an understanding of the degree of access, confidence, skills, and the influence and use of evidence across a sample of LGs in Victoria, it did not address the individual experiences and contextual barriers of evidence use and general decision-making processes. A qualitative study using in-depth interviews was conducted to explore the processes associated with EIDM in LGs. All EviDenT participants were invited to participate in interviews, 98 volunteered, 19 participants were selected, and 13 interviews were conducted. Interviewees were sampled according to a matrix to ensure a mix of professional backgrounds (*e.g.*, health promotion, environmental health, planning) and position titles (*e.g.*, CEO, managers, project officers). The interviews were based on three core questions on defining evidence, practising EIPH, and processes for EIPH: how do you define evidence? Is this different from how evidence is perceived in your organization? What are typical example/s of decisions and attempt to track evidence-informed process?; What strategies would support EIDM in your council? This final question encouraged LGs to help to create the design of the intervention, and as such the intervention was viewed very much as one designed in partnership. Key themes emerged related to defining evidence, how evidence was being used generally, and contextual influences upon evidence use. Thematic analysis identified potential strategies that would be most useful to facilitate EIDM in the contexts of participants interviewed. These are described below.

### Skills development training

Training sessions targeted to both project officers and senior management were highlighted as a key strategy to support EIDM. Suggested content included how to access research evidence, updates on current intervention research, and the research process (to support understanding of how information gathering in a LG context could or should occur). Frequency and duration of training was discussed, as turnover in LG staff would prevent one-off workforce development sessions from impacting on EIDM. Search skills for accessing evidence were called for given the variability in familiarity with Internet repositories between individuals.

### Resources and tools for decision making

Tools to support EIDM were proposed, reflecting a need to support understanding in assessing trustworthiness of evidence, and applying evidence to decision making; as was guidance on where evidence could be used in the stages of decision-making processes, how different sources of evidence can be combined, and access to tailored resources, such as evidence summaries.

### Networking for information sharing

A range of networks already existed that supported LG staff, however networks with external input (*e.g.*, other government agencies, academics) were considered to be more useful in promoting evidence sharing. The seniority of individuals attending networking opportunities also appeared to be a consideration if networking was to be a KT strategy. Support from senior management was recognized as important to ensure networking opportunities drive action. Participants acknowledged the importance of relationships with researchers, although the relevance of research and understanding of LGs purpose and context for evidence use needed to be understood. Access to external support was perceived to be potentially useful (including assisting with access and making sense of research evidence). This may also facilitate skill development.

### Development of an intervention logic model

**Figure 1:**
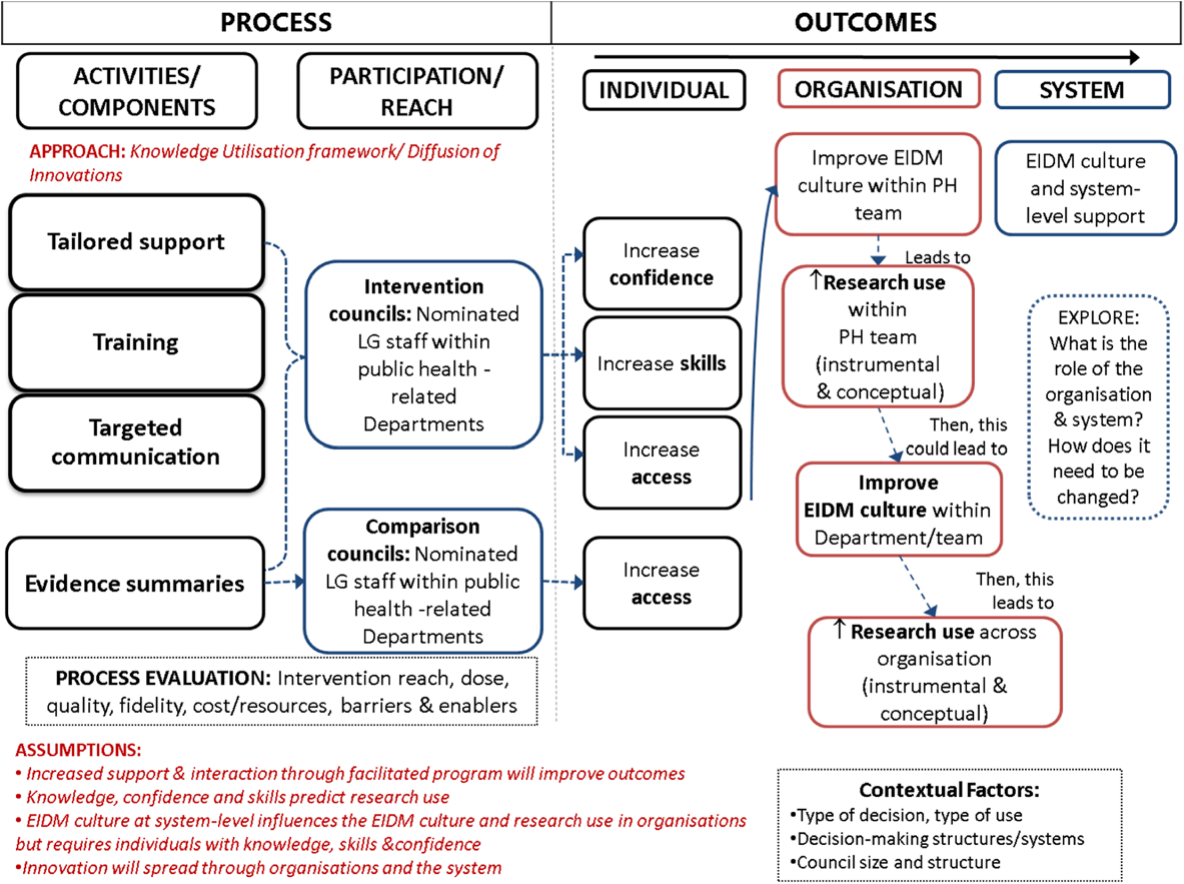
Logic model: KT intervention for LG.

The preliminary studies described above informed the development of a logic model (see Figure 1), which identifies the processes and pathways by which the multi-strategy KT would be implemented. A logic model is a depiction of a system within which an intervention fits and identifies core elements and relationships that operate within that system. Logic models have been applied to programs and interventions to identify how they might work and how problems may be solved. Logic models make explicit the underlying components of a program and any underlying assumptions [50-53].

The logic model for KT4LG was underpinned by diffusion of innovations theory. Diffusion of Innovations theory was used to identify the points at which a KT intervention could facilitate EIDM [21,54,55]. It was also used to explore how ideas are shared (at process and outcome level). In addition, knowledge management theory and research utilization theory were applied to provide perspectives on how evidence may be used in an organizational context [56-59].

KT frameworks (often based on diffusion of innovations theory) were also used to inform the intervention design. In particular, the framework developed by Bowen and Zwi describes the importance of policy processes, such as the policies influencing current planning within LGs, and the ways in which evidence can be sourced, used, and applied [4]. Given the absence of documented organizational factors such as decision-making influences, staff capacity and strengths, and the sheer variability in organizational and geographical location, it was envisaged that KT4LG needed to commence with engagement of individuals within the organizations to identify the most potent organizational- and system-level requirements for organizational change.

## Results and discussion

### Intervention aims

**Figure 2:**
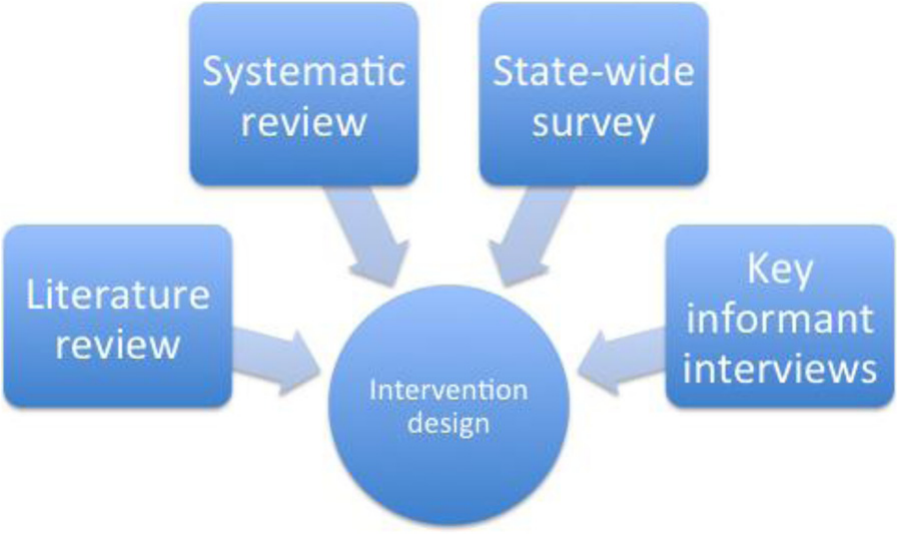
Preliminary studies and their contribution to intervention design.

We integrated the findings from the preliminary research studies (Figure 2) to inform the intervention design and associated implementation plan. Given that previous KT intervention research has tested strategies such as an online repository of public health evidence (health-evidence.ca [60]) and knowledge brokering [61], and given the strong preference for support strategies and workforce development [62,63], a multi-strategy KT capacity building intervention was proposed.

The overall aims of the intervention were:

1. To increase access to research evidence through provision of evidence summaries and additional individualized support, such as tailored messages.

2. To develop skills in accessing research, assessing trustworthiness, and applying research evidence to local context.

3. To develop and implement strategies that assist in the development of an organizational culture that supports EIDM within LGs.

Figure 1 represents the logic model developed to describe intervention design and to guide the implementation plan. Based on the findings of the preliminary studies, the intervention would comprise three core components: tailored organizational support, group training, and targeted messages and evidence summaries. All comparison councils would have access to the evidence summaries.

### Intervention implementation

The components of the intervention were integrated and delivered continuously across two years, based on reflections from previous research for a longer implementation period [47].

The intervention was designed to be implemented by a Program Coordinator (RCl and then subsequently TP), who would also provide a point-of-contact and act as a KB. The term ‘program’ was used as it was deemed more appropriate for use with the target population. While the overall priority content area was obesity prevention, the intervention and skills required of the Program Coordinator needed to be wider in order to meet the demands of public health practitioners in the LG sector. Given the variation in LG size, the numbers of invited participants from each council would vary but were limited to four.

### Tailored organizational support

Monthly one-on-one contact with staff in councils would occur via telephone, initiated by the Program Coordinator. This could be more or less frequent depending on participant needs. Phone calls will focus on public health related projects, ideally with a focus on obesity prevention. The Program Coordinator planned to identify how evidence could be further incorporated at various stages of the program planning, implementation, and evaluation cycle. Barriers and facilitators to EIDM will be discussed in order to provide the Program Coordinator with information to tailor support. Assessment of need for tailored support would be conducted at the initial and mid-intervention visits.

The preliminary studies and review of theoretical perspectives recommended the inclusion of site visits, highlighting the potential usefulness of enhanced interaction between researchers/knowledge brokers and the individuals/agencies. A mid-term site visit was therefore proposed for each LG. The objectives of the visits would be agreed mutually by the LG and Program Coordinator, and could include activities such as group training, working meetings, or presentations. Site visits may also be used to discuss individual and organizational culture for use of research evidence among the public health team, guided by self-assessment tools developed and evaluated by the Canadian Health Services Research Foundation [64,65]. Strategies to address organizational evidence use were an exploratory component of the intervention.

### Group training

Group training would be held biannually in a central location. A modular approach was planned to cover core principles of evidence-informed public health asking answerable questions, searching, trustworthiness, assessing applicability and transferability, and evaluation; with a range of activities designed to encourage networking between councils. It was anticipated that participants would contribute to the focus of these sessions. Shared development of the logic model identified the need for networking sessions, which would be incorporated into the group training sessions rather than run separately as it was felt that LG staff may already be participants in formal networks and additional networks may prove too time consuming.

Mid-intervention council visits would be used to examine the fidelity of the intervention, and to assess the context of EIDM within each council. Council-based training would be offered once, at the mid-point of the intervention, as a mechanism to engage a wider audience (increase within-council reach) and to meet the needs of the council, particularly if geographic distance is a barrier to regular attendance at central training sessions.

### Targeted communication and evidence summaries

Evidence summaries, outlining possible obesity prevention intervention options, were to be developed in partnership with participants and other LG contacts across Australia. Legislative requirements of councils would be explored and considered, and relevance to the role of councils in service delivery and program implementation incorporated. The Program Coordinator would work in partnership with councils to develop these documents prior to distribution to ensure appropriateness and relevance of content. Evidence summaries would be available to all LGs. These summaries would be used to promote evidence-informed options within intervention LGs. In addition, the Program Coordinator would send intervention LGs targeted communication that may include newly published studies or reviews, and upcoming conferences and training opportunities.

### Intervention outcomes

As the logic model proposes, a range of outcomes would be possible at the individual-, organizational-, and system-levels. The KT4LG intervention study [66] sought to measure the impact of the KT intervention on individuals’ confidence, skills, and access to research evidence compared to a control group not receiving the same intervention. Further, KT4LG attempted to assess changes in the organizational culture for EIDM, with the expectation that if this occurs, then research use (instrumental and conceptual) would increase among public health teams, which ultimately could be expected to increase research use across the organization and the system within which LG operates.

### Limitations

The design of the intervention is limited primarily to the implementation of KT interventions targeted at individuals. The logic model demonstrates how individual interventions may be linked to organizational- and system-level interventions. Given the paucity of literature on interventions directed at these levels with the intent of increasing organizational culture we will explore potential strategies during the implementation of KT4LG.

The size and scope of LG was identified as an issue for the implementation of KT4LG in the preliminary studies—particularly the qualitative study. It was anticipated that participating in this intervention might be more challenging for LGs with smaller staffing levels, smaller budgets, and/or limited research capacity.

The design of KT4LG revealed the challenges in measuring outcomes at both individual-, organizational-, and system-levels. Based on the results of the preliminary studies the research team chose to focus on three core domains: access, confidence, and organizational culture. There was limited evidence on which to base this decision. The implementation and evaluation of KT4LG will reveal more about the usefulness of these domains.

## Conclusions

KT4LG aims to respond to the lack of rigorous evidence to guide KT in public health decision-making contexts. Preliminary research informing the intervention included a review of theoretical and narrative literature, a systematic review of the effects of KT strategies, a survey of LGs in Victoria, and a qualitative study exploring decision-making processes in this setting. The KT4LG intervention was designed using a partnership approach and involved an international research team interested in the development and mixed method evaluation of innovative and evidence-based complex interventions and practitioners in LG public health. As such, KT4LG aimed to co-generate new, practical evidence on what works to support EIDM in the LG context. The intervention design was informed by diffusion of innovations theory, research utilization theory, and KT frameworks. The intervention outlined in the logic model was designed to address contemporary demands including: imposed policy expectations for the use of evidence in policy and planning; organizational demands for best available evidence; identification of factors for system-wide solutions; opportunities to embed KT competencies into planning and employment; the need for increased access to relevant research; skills to interpret and use research; and network building. Guided by the overarching logic model, intervention components included selection of participants involved in key public health decision-making contexts, group training and networking sessions, monthly contact with a Program Coordinator, and relevant evidence summaries.

The interest in ‘what works for whom and why’ about public health issues such as obesity prevention while addressing trustworthiness and relevance of research evidence, is compelling and a great drawcard for a new, realistic approach to public health research and evidence-informed public health in particular. Answering questions of relevance and priority can help bridge the evidence-practice gap. KT4LG is an example of a facilitated KT intervention that presents an approach designed to increase the use of research evidence in policy and practice.

## Abbreviations

APAIS: Australian public affairs information service; CINAHL: Cumulative index to nursing and allied health literature; EIDM: Evidence-informed decision-making; KB: Knowledge broker; KT: Knowledge translation; KT4LG: Knowledge translation for local government; LG: Local government.

## Competing interests

The authors declare that they have no competing interests.

## Authors’ contributions

All authors were actively involved in the development and design of the intervention and study. RA drafted the initial manuscript. EW is the principal Chief Investigator and led the study, RA was the project manager, RCl and TP coordinated intervention activities and in this capacity RCl contributed to the logic model development. All authors contributed to the draft of this paper and signed off on the final version.
